# RNA Regulated Toxin-Antitoxin Systems in Pathogenic Bacteria

**DOI:** 10.3389/fcimb.2021.661026

**Published:** 2021-05-18

**Authors:** David D. Sarpong, Erin R. Murphy

**Affiliations:** ^1^ Department of Biological Sciences, Ohio University, Athens, OH, United States; ^2^ Infectious and Tropical Diseases Institute, Ohio University, Athens, OH, United States; ^3^ Molecular and Cellular Biology Program, Ohio University, Athens, OH, United States; ^4^ Department of Biomedical Sciences, Ohio University, Heritage College of Osteopathic Medicine, Athens, OH, United States

**Keywords:** sRNA, pathogen, bacteria, RNA, toxin-antitoxin (TA)

## Abstract

The dynamic host environment presents a significant hurdle that pathogenic bacteria must overcome to survive and cause diseases. Consequently, these organisms have evolved molecular mechanisms to facilitate adaptation to environmental changes within the infected host. Small RNAs (sRNAs) have been implicated as critical regulators of numerous pathways and systems in pathogenic bacteria, including that of bacterial Toxin-Antitoxin (TA) systems. TA systems are typically composed of two factors, a stable toxin, and a labile antitoxin which functions to protect against the potentially deleterious activity of the associated toxin. Of the six classes of bacterial TA systems characterized to date, the toxin component is always a protein. Type I and Type III TA systems are unique in that the antitoxin in these systems is an RNA molecule, whereas the antitoxin in all other TA systems is a protein. Though hotly debated, the involvement of TA systems in bacterial physiology is recognized by several studies, with the Type II TA system being the most extensively studied to date. This review focuses on RNA-regulated TA systems, highlighting the role of Type I and Type III TA systems in several pathogenic bacteria.

## Introduction

At each point in the transmission and infection cycle, pathogenic bacteria are presented with various, and often extreme environmental conditions to which they must quickly adapt to survive. Their rapid response to environmental change allows bacteria to survive under the harshest of conditions ranging from nutritional stress, extreme temperatures, changing osmolarity, alterations in pH, and even threats of attacks from viral predators (bacteriophages) and the host immune response (Fernández et al., 2018). Pathogenic bacteria have evolved a variety of mechanisms by which to adapt to environmental changes, many of which involve protein factors that regulate the expression of a specific gene(s) in response to specific environmental signals (Brooks et al., 2011). An increasing number of studies, however, have revealed the vast potential of small RNAs (sRNAs) in controlling complex regulatory circuits connected to bacterial survival and/or virulence (reviewed in Plaza and José, 2020). One role that sRNAs play in controlling bacterial physiology and virulence is that of the anti-toxin component of Type I and Type III Toxin-Antitoxin (TA) systems (Brantl and Jahn, 2015; Holmqvist and Wagner, 2017).

## Classification of Bacterial Toxin-Antitoxin Systems

TA systems are composed of two components: a stable toxin, and an unstable antitoxin which represses the synthesis or activity of the associated toxin (Harms et al., 2018). TA systems are categorized into six different types (Types I-VI) based on the nature and regulatory action of the antitoxin component (Page and Peti, 2016). Whereas the toxin of a TA system is always a protein, the antitoxin is either a protein (Types II, IV-VI) or an sRNA (Types I and III) (Unterholzner et al., 2013; Harms et al., 2018). Specifically, Type I and III TA systems involve sRNA antitoxins which either repress the translation (Type I) or activity (Type III) of the associated toxin protein. The Type II TA system is the most studied and involves a protein antitoxin which neutralizes the cognate toxin protein through protein-protein interactions. Antitoxin proteins of Type IV TA systems disrupt the activity of the associated toxin indirectly by competitively interfering with binding to their cellular targets. Type V antitoxin is an RNase that represses its cognate toxin at the level of transcription by cleaving the mRNA molecule on which the toxin is encoded. Finally, the newly discovered Type VI TA system involves an antitoxin protein which promotes toxin degradation by recruiting a protease to cleave it (Unterholzner et al., 2013; Harms et al., 2018; Song and Wood, 2020b).

## Type I and Type III TA Systems in Pathogenic Bacteria

Numerous studies have linked bacteria survival and pathogenesis to TA systems (Lobato-Márquez et al., 2015; Fernández-García et al., 2016; Lobato-Márquez et al., 2016a). Most of these studies focus on Type II TA systems (Korch et al., 2009; Zhu et al., 2009; Heaton et al., 2012; Wang et al., 2015). In this review, we highlight Type I and Type III TA systems and the role they play in the survival, pathogenicity, and persistence of select bacterial pathogens.

## Section 1: Type I Toxin Antitoxin Systems

### Type I TA Toxins Target Various Important Cellular Components and Produce Different Effects

TA system toxins target important cellular components of the bacteria producing it, affecting vital physiological processes such as ATP synthesis, DNA replication, transcription, translation, and cell wall synthesis (Unterholzner et al., 2013; Harms et al., 2018; Jurėnas and Van Melderen, 2020). It is this activity that ultimately leads to toxin-associated bacterial growth inhibition or lysis.

Toxins from Type I TA systems are typically small (<60 aa) hydrophobic transmembrane proteins, (Fozo et al., 2008a). The toxic activity of these small proteins varies (Brielle et al., 2016), however the lethal effect of most Type I toxins is mediated by pore formation in the bacterial membrane, leading to disruption of membrane integrity, membrane depolarization, and subsequent ATP depletion (Wen and Fozo, 2014). This toxic mechanism is exemplified by the TisB, DinQ and HoK toxins of *Escherichia coli* (Wen and Fozo, 2014), as well as the SprG1, PepA1 and PepA2 toxins of *S. aureus* (Pinel-Marie et al., 2014; Germain-Amiot et al., 2019). Rather than disrupting the membrane potential, the BsrG toxin of *B. subtilis* and the AapA1 toxin of *Helicobacter pylori* target cell envelope synthesis directly, resulting in the formation of invaginations within the cell and ultimately cell lysis (Jahn et al., 2015; El Mortaji et al., 2020). Other Type I TA system toxins mediate their lethal effect via endoribonuclease activity (Kawano, 2012), endodeoxyribonuclease activity (Guo et al., 2014) or by promoting nucleoid condensation (Weaver, 2020). These toxic activities are exemplified by the toxin component of the *E. coli* SymE-SymR TA system, the *E. coli* RalR-RalA TA system, and the *Enterococcus faecalis* Fst-RNAII TA system, respectively (Kawano, 2012; Guo et al., 2014; Weaver, 2020). The activity, target(s) and function of select Type I and Type III toxin is summarized in **Table 1**.

**Table 1:** Summary of the activity, target and function of select toxins produced by Type I and Type III TA systems in pathogenic bacteria.

Toxin	Pathogen	Toxin Activity/Target	Function	References
SprG1	*S. aureus*	Pore forming toxin/Membrane	Virulence, Competition	Pinel-Marie et al., 2014
PepA1	*S. aureus*	Pore forming toxin/Membrane	Virulence, Competition	Sayed et al., 2012b
PepA2	*S. aureus*	Pore forming toxin/Membrane	Virulence, Competition	Germain-Amiot et al., 2019
BsrG	*B. subtilis*	Affects cell wall formation/Cell wall machinery	Unknown/Chromosomal element stability(?)	Jahn et al., 2012
_1_TisB _2_TisB_ST_	_1_ *E. coli* *_2_S.* Typhimurium	Pore forming toxin/Membrane	_1_Persistence _2_Survival in macrophage	_1_ Dörr et al., 2010 _2_ Lobato-Márquez et al., 2015
ZorO	*E. coli*	Pore forming toxin(?)/Membrane	Unknown	Wen et al., 2017
_1_Hok _2_HokB _3_Hok_ST_	_1,2_ *E. coli* _3_ *S.* Typhimurium	Pore forming toxin/Membrane	_1_Plasmid maintenance _2_Persister _3_Survival in macrophage	_1_ Gerdes et al., 1986b _2_ Verstraeten et al., 2015; Wilmaerts et al., 2018; Wilmaerts et al., 2019 _3_ Lobato-Márquez et al., 2015
_1_LdrD _2_LdrA_ST_	_1_ *E. coli* _2_ *S.* Typhimurium	Nucleoid condensation/Nucleoid	_2_Survival in macrophage	_1_ Kawano, 2012 _2_ Lobato-Márquez et al., 2015
SymE	*E. coli*	Endoribonuclease/RNA	SOS Response/Recycling of Damaged RNAs	Kawano, 2012
RalR	*E. coli*	Endodeoxyribonuclease/DNA	Survives Fosfomycin	Guo et al., 2014.
_1_Fst _2_Fst_sm_	_1_ *E. faecalis* _2_ *S. mutans*	Nucleoid condensation/Nucleoid	_1_Plasmid addiction _2_Reduced persistors	_1_ Weaver et al., 1996; Greenfield et al., 2001; Weaver et al., 2009 _2_ Koyanagi and Lévesque, 2013
AapA1	*H. pylori*	Coccoid shape formation/Membrane	Unknown/Stress induced dormancy(?)	El Mortaji et al., 2020
_1_TimP _2_RyfA	_1_ *S.* Typhimurium _2_ *S. dysenteriae*	_1_Membrane protein/Membrane Membrane _2_protein(?)/Membrane(?)	_1_Unknown _2_Virulence	_1_ Andresen et al., 2020 _2_ Fris et al., 2017

(?) denotes a function that is proposed but not yet experimentally validated.

### Type I TA System Antitoxins Are Regulatory sRNA Molecules

The antitoxin within Type I TA systems is an sRNA that represses production of its cognate toxin through complementary base paring to the toxin encoding transcript, leading to toxin mRNA degradation, translation inhibition, or both (Wen and Fozo, 2014; Brantl and Jahn, 2015). The genes encoding each component of a Type I TA system are encoded within a single locus, and in many cases the antitoxin is encoded antisense (*cis*) to the toxin (Brielle et al., 2016). Some antitoxins, however, are encoded adjacent to, or partially overlapping with, the coding region of the toxin (*trans*) in either a divergent, convergent or parallel orientation (Fozo et al., 2008b). Type I TA systems of most Gram-positive bacteria have toxin and antitoxin genes arranged antisense to each other with partial overlap at their 3’ ends, resulting in complete complementarity between the corresponding areas of the antitoxin sRNA and toxin transcript (Masachis and Darfeuille, 2018; Brantl and Müller, 2019). Interestingly, the functional interaction between these antitoxin sRNAs and their target toxin transcript, however, does not always occur via these complementary sequences. For example, in *Staphylococcus aureus sprA2*/SprA2_AS_ and *sprA1*/SprA1_AS_ Type I TAs, though the 3’ ends of the toxin and antitoxin genes overlap, antitoxin-mediated regulation occurs via interactions between the 5’ end of the molecule (Sayed et al., 2012a; Germain-Amiot et al., 2019).

**Figure 1:**
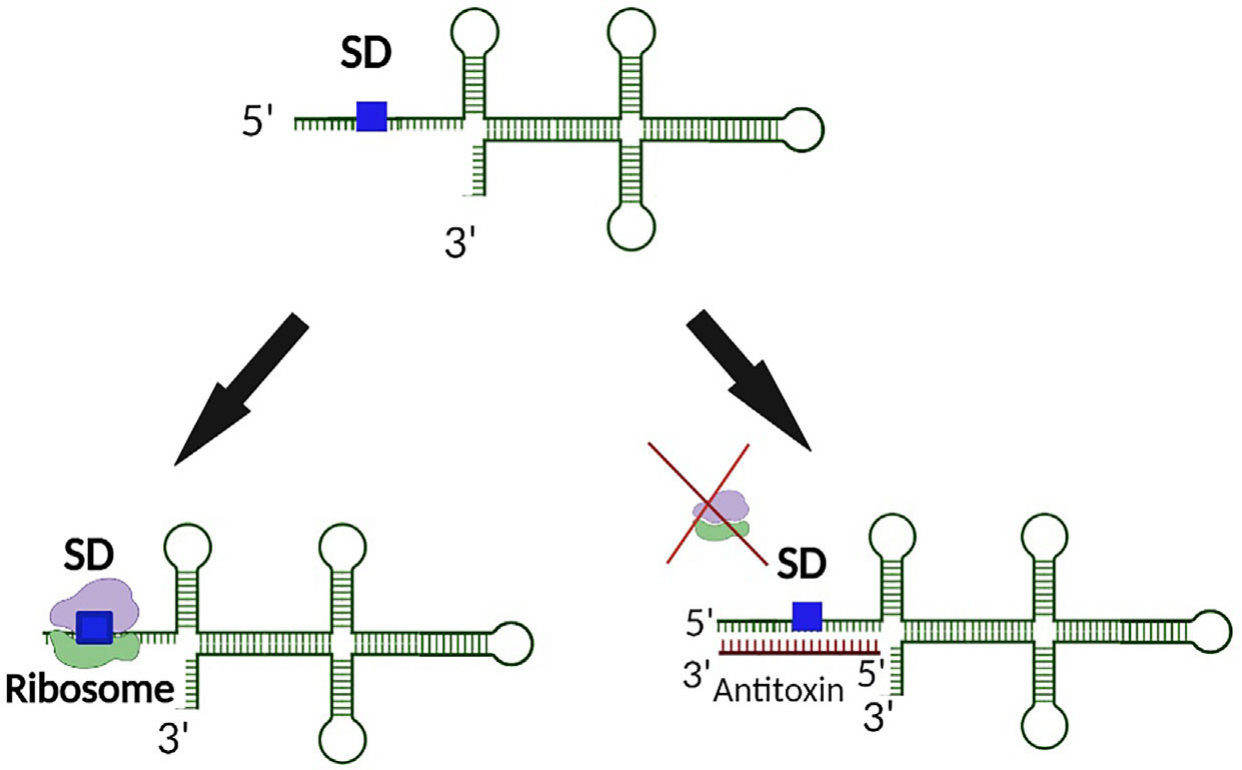
Binding of a Type I antitoxin occludes the SD site on the toxin transcript. In a typical mechanism of regulation, the antitoxin of a Type I TA systems binds the target toxin transcript such that binding of the ribosome to the SD site is physically blocked. As such, translation of the Type I toxin is prevented.

**Figure 2:**
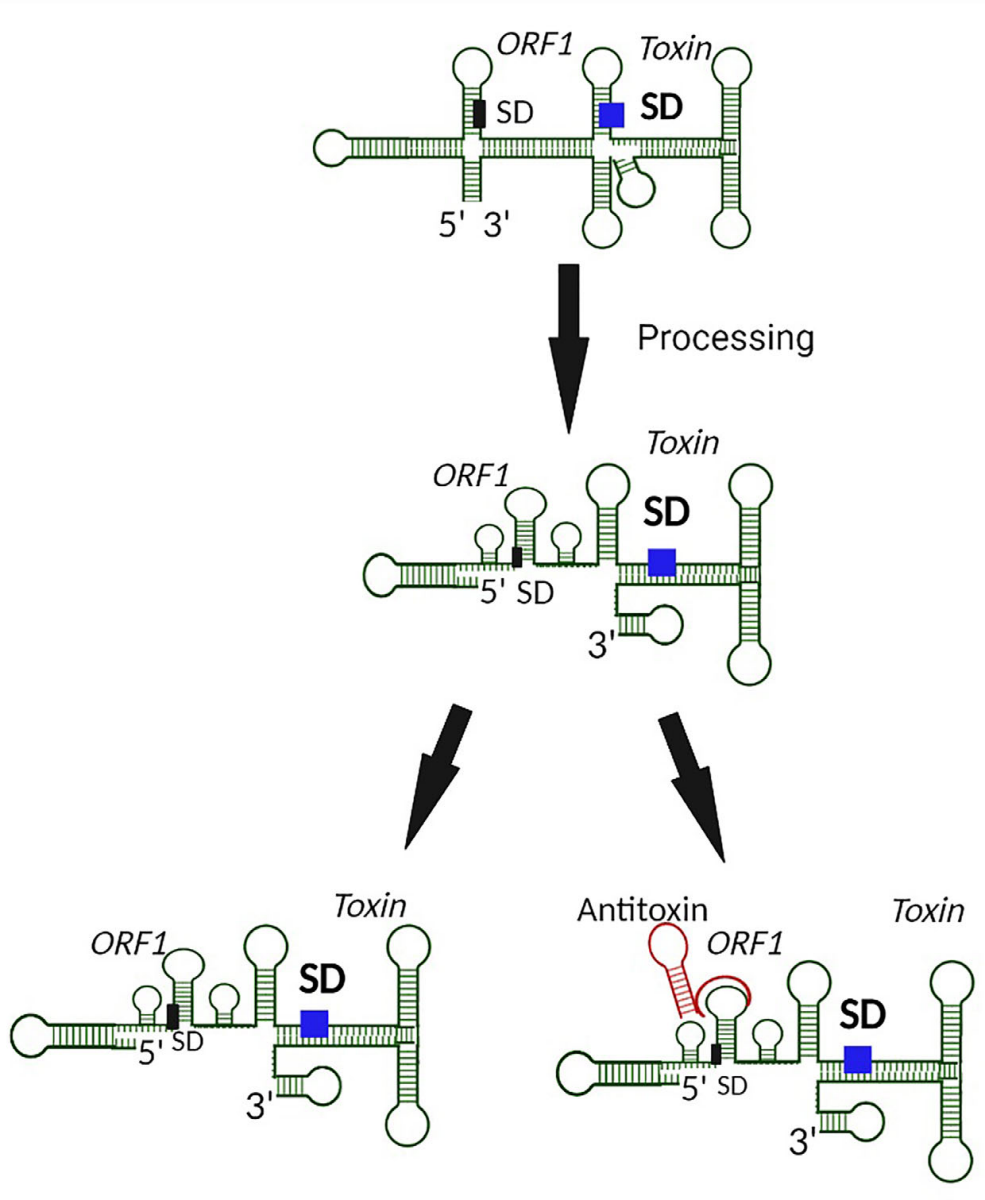
Prevention of Type I toxin production by blocking of an upstream coupled open reading frame by the antitoxin. In the case where translation of a toxin is coupled to translation of an upstream open reading frame (ORF1), binding of the antitoxin to the upstream SD prevents translation from both open reading frames, including from that encoded Type I toxin.

**Figure 3:**
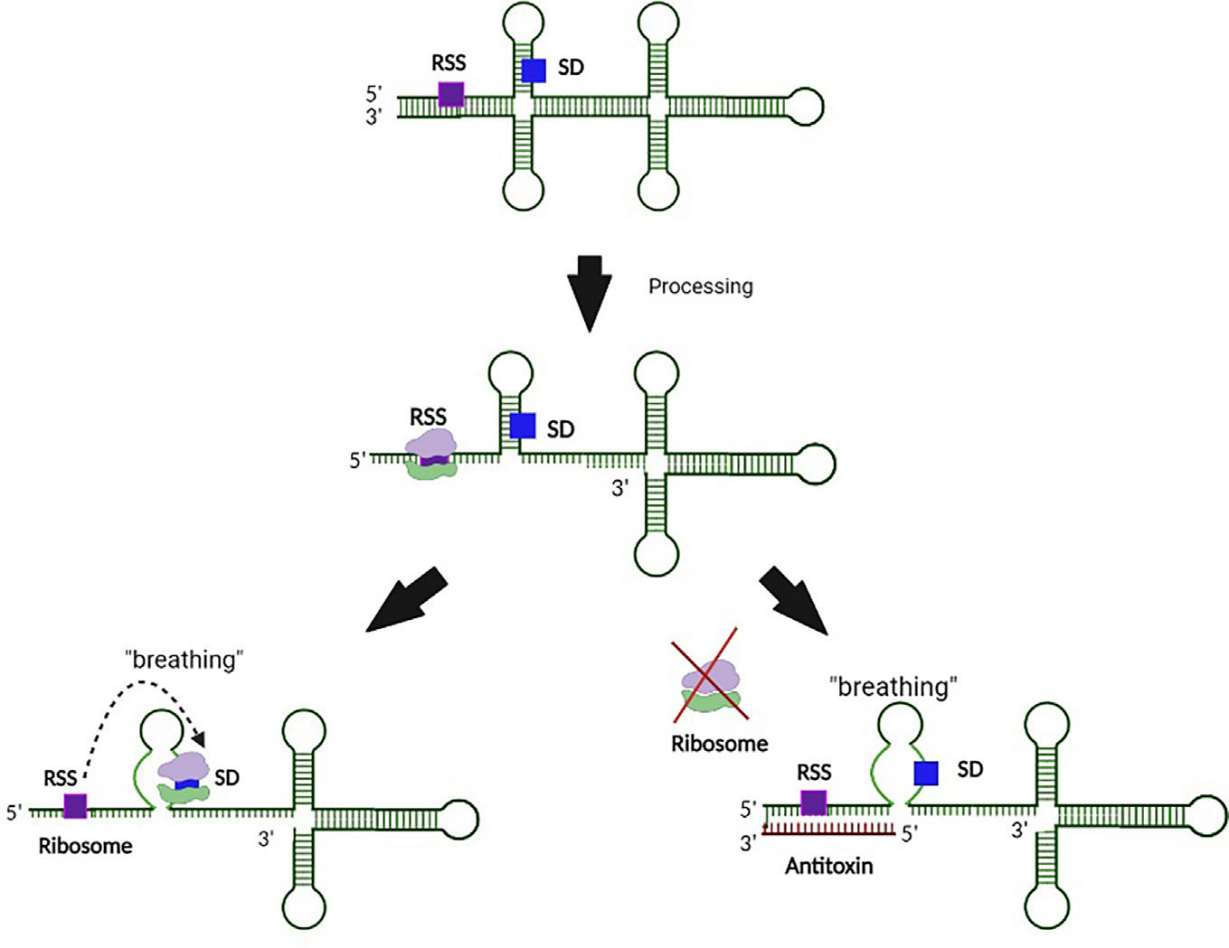
Regulation of Type I toxin production by binding of the antitoxin to an associated ribosomal standby site. In this example, processing of the toxin transcript exposes a ribosomal standby site (RSS) that facilitates binding of the ribosome to the SD site. Interaction between the antitoxin and RSS on the toxin transcript prevents ribosomal binding to this site, and thus subsequent binding to the SD. Such regulation prevents translation of the Type I toxin.

Functional regulation by the sRNA antitoxin is often mediated by binding between partially complementary nucleic acid sequences of the antitoxin sRNA and the target toxin transcript that results in occlusion of the Shine Dalgarno (SD) site on the toxin transcript, and thus inhibition of translation and/or degradation of the transcript (**Figure 1**) (Wen and Fozo, 2014). Alternatively, binding of a Type I antitoxin sRNA can occur at the 3’ end or elsewhere within the toxin transcript, generating an sRNA-mRNA heteroduplex that is cleaved by an RNase (Durand et al., 2012a; Wen and Fozo, 2014). Of note, the interaction between an sRNA antitoxin and target toxin transcript can lead to both translation inhibition and transcript degradation (Jahn et al., 2012; Brantl and Müller, 2019). Another mechanism by which Type I TA antitoxins regulate toxin production is to block a reading frame whose translation is coupled to that of the toxin gene (**Figure 2**) (Thisted et al., 1994). Finally, rather than preventing translation by binding directly to the SD of their target toxin mRNA, some Type I antitoxins achieve this end by binding to a ribosome standby site required for translation of the toxin (**Figure 3**) (Darfeuille et al., 2007).

### Hfq Is Not Required for the Activity of All Type I TA Antitoxin RNAs

While many Type I sRNA antitoxins are encoded antisense to their target transcript, and thus have an extensive amount of perfect sequence complementarity with their cognate toxin transcript, those encoded in *trans* often display limited complementarity to their targets (Fozo et al., 2008b). Many bacterial sRNAs that share limited nucleic acid complementarity with their target mRNAs require a protein chaperone such as Hfq to facilitate regulation (Brennan and Link, 2007; Waters and Storz, 2009). Interestingly, the regulatory function of antitoxin sRNAs, even for those with limited nucleic acid complementary to their cognate toxin transcript, such as TisB/IstR-1, ShoB/OhsC, ZorO/OrzO in *E. coli* (Unoson and Wagner, 2008; Fozo, 2012; Wen et al., 2014) or BsrG/SR4, BsrE/SR5, *yonT*/SR6 (Jahn et al., 2012; Müller et al., 2016; Reif et al., 2018), does not require Hfq (Dambach et al., 2013). A notable exception to this rule is the sRNA antitoxin RalA of the *E. coli* RalR/RalA Type I TA system (Guo et al., 2014). Additionally, though the SR5 Type I antitoxin of *B. subtilis* is stabilized by Hfq, its regulatory activity can occur in its absence (Müller et al., 2016).

### Additional Modes of Toxin Regulation by Type I Antitoxin RNA Molecules

**Figure 4:**
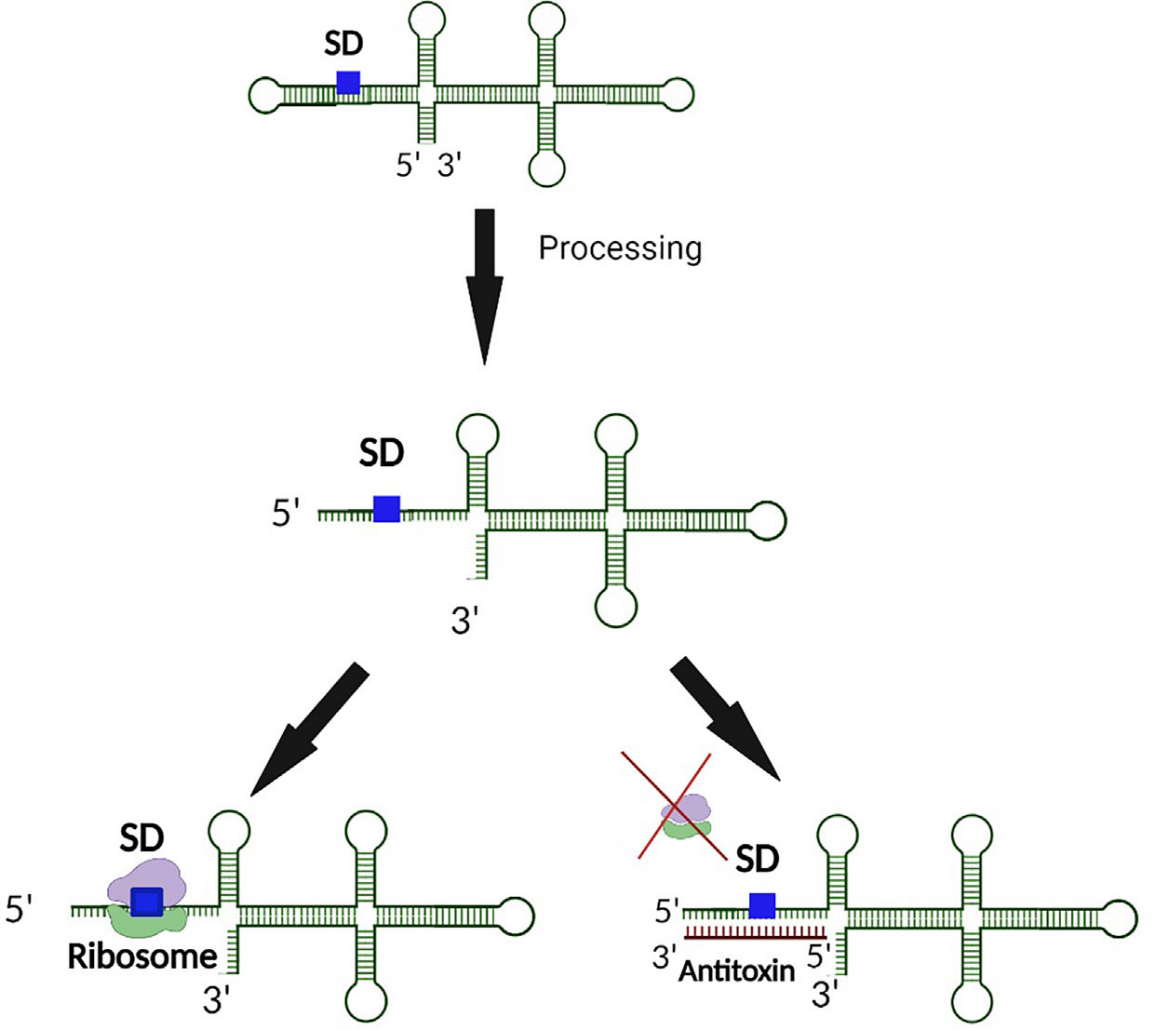
5’ end processing represents a second layer of regulation controlling the production of some Type I toxins. In the event an inhibitory secondary structure exists at the 5’ end of the toxin transcript, 5’ end processing exposes both the SD and antitoxin binding site. Upon exposure of the binding region, translation of the toxin is further regulated by an interaction between the antitoxin and the SD of the toxin transcript such that ribosomal binding is inhibited. Such dual regulation ensures minimal production of the Type I toxin when its activity would be deleterious to the bacterium.

**Figure 5:**
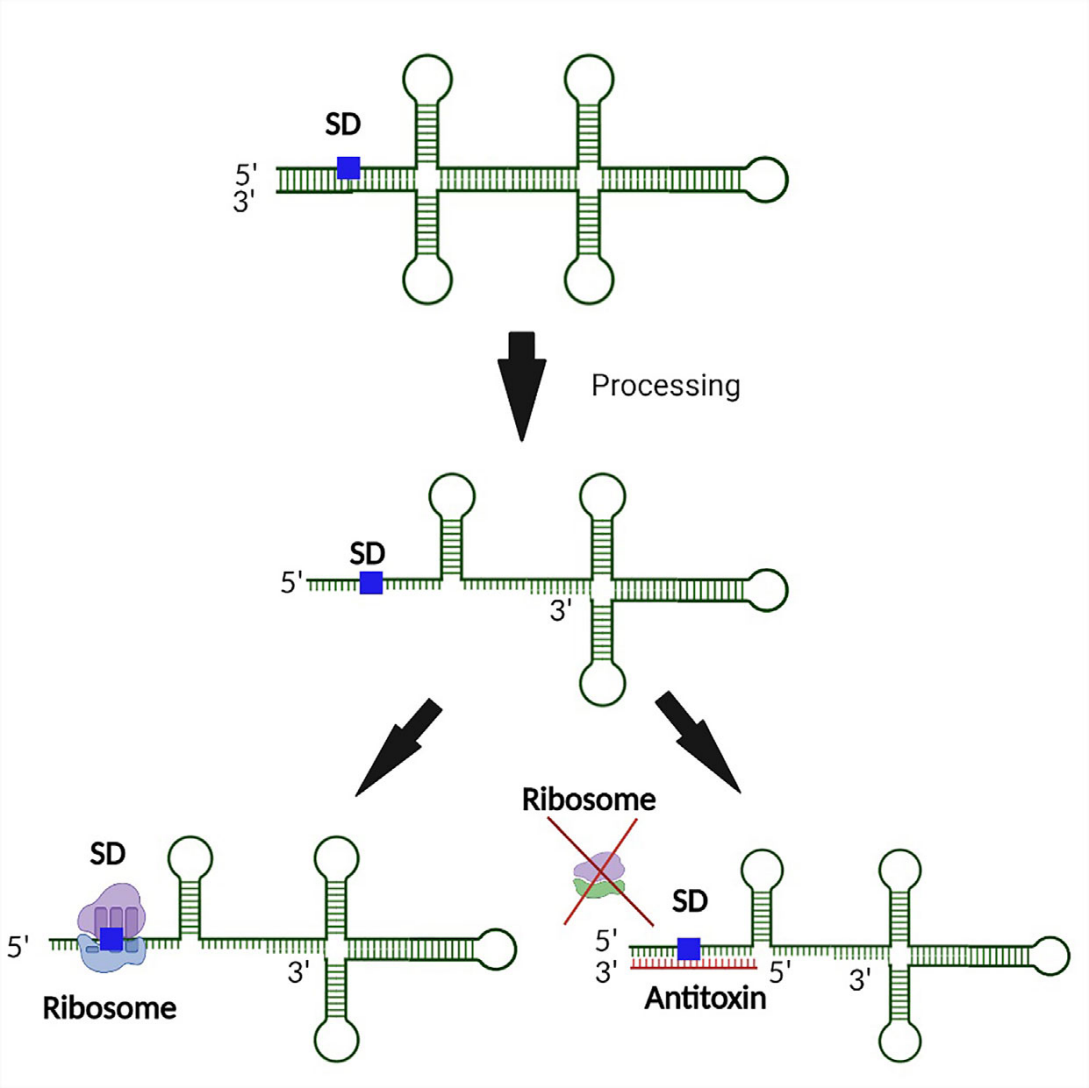
3’ end processing represents a second layer of regulation controlling the production of some Type I toxins. In the event an inhibitory secondary structure formed by binding between the 5’ and 3’ end of the toxin transcript, 5’ end processing is required to expose both the SD and antitoxin binding site. Upon exposure of the binding region, translation of the toxin is further regulated by an interaction between the antitoxin and the SD of the toxin transcript such that ribosomal binding is inhibited. Such dual regulation ensures minimal production of the Type I toxin when its activity would be deleterious to the bacterium.

**Figure 6:**
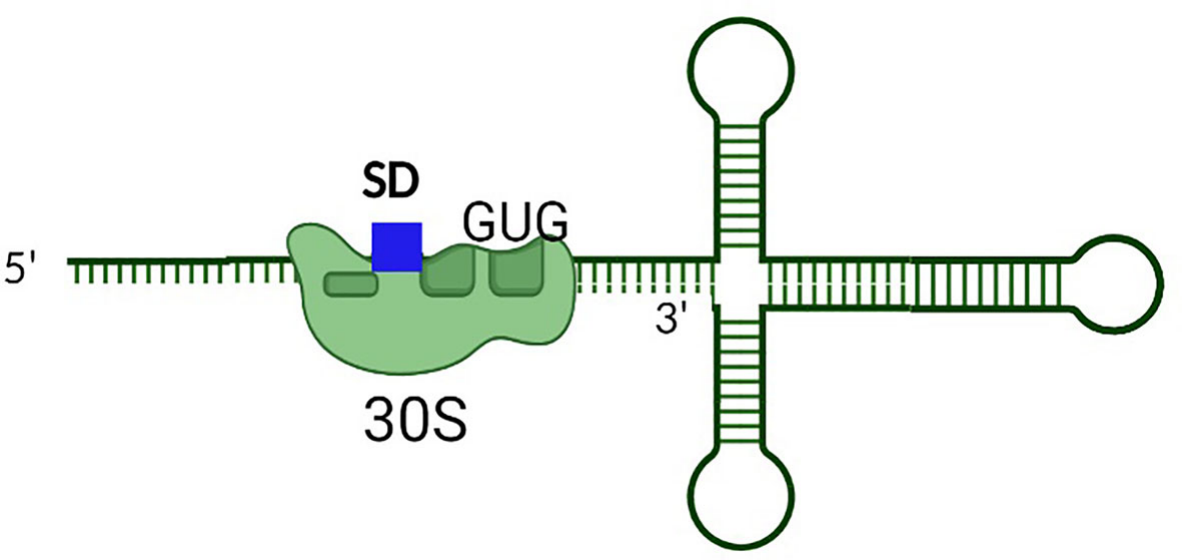
The presence of a non-AUG translational start codon is a secondary mechanism to limit the production of some Type I toxins. In this example, the presence of the non-AUG translational start codon results in partial inhibition of translation from the toxin transcript. Such regulation limits the production of Type I toxins when its activity would be deleterious to the bacterium.

**Figure 7:**
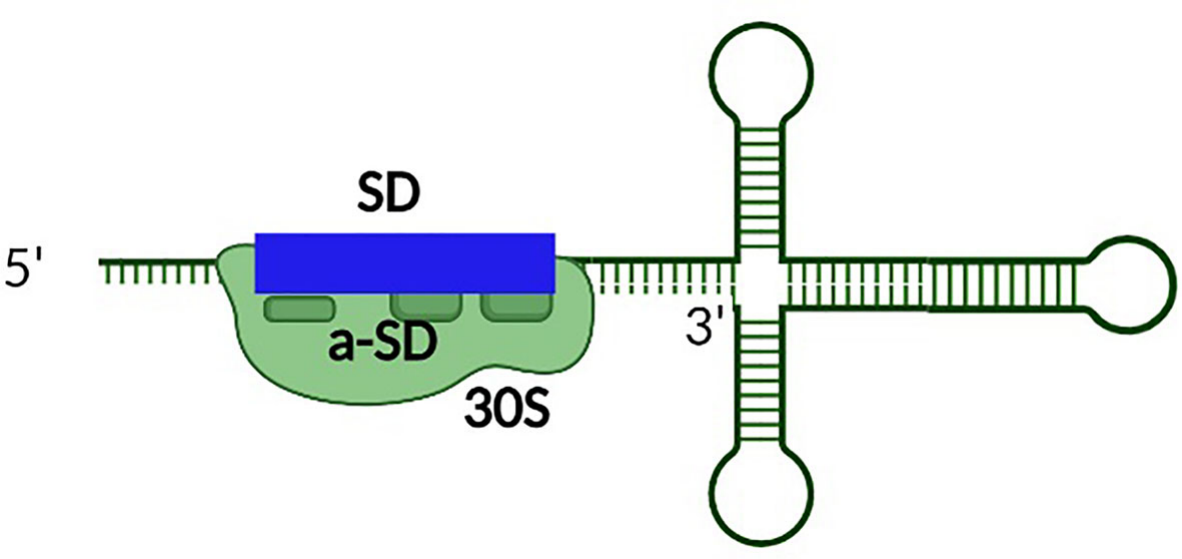
The presence of perfect complementarity between the SD site of the toxin transcript and the anti-SD sequence within the rRNA represses the production of some Type I toxins. Unusually tight binding between the SD site and anti-SD site within the rRNA of the ribosome reduces toxin production by inducing ribosomal pausing.

Premature synthesis of the toxin in the absence or low abundance of the corresponding antitoxin would be deleterious to bacterial survival. Hence additional steps of regulation are required to prevent such premature toxin production. To this end, secondary layers of toxin regulation which transcend the simplistic view of antitoxin regulation limited to complementary base-pairing between the toxin mRNA and antitoxin sRNA have evolved (Masachis and Darfeuille, 2018). In these instances, transcription of the toxin gene is uncoupled from its translation via mechanisms such as the sequestration of the ribosomal binding site within a hairpin structure in the 5’ untranslated region (Weaver et al., 2009; Durand et al., 2012a; Sayed et al., 2012a; Kristiansen et al., 2016; Müller et al., 2016; Wen et al., 2017; Masachis and Darfeuille, 2018). Translation of the toxin, and in some cases, interaction of the toxin with the antitoxin then requires processing of the transcript at either the 5’ or 3’ end by ribonucleases (Wen and Fozo, 2014; Masachis et al., 2019). Such processing results in a structural alteration of the toxin transcript which exposes the SD, thus facilitating translation and, potentially, interaction with the antitoxin (**Figure 4**). In another example, a sequence complementary to the Shine Dalgarno sequence (anti SD) is located within the 3’ end of the toxin transcript. Such complementarity results in the formation of a 5’-3’ long distance interaction (LDI) that inhibits translation by occluding the SD (Arnion et al., 2017; Masachis et al., 2019). Once again, processing of the transcript is required for binding of the transcript by the ribosome or antitoxin (**Figure 5**). Finally, in some cases the rate of toxin translation is reduced by the presence of a non-canonical start codon (**Figure 6**) (Durand et al., 2012b; Masachis and Darfeuille, 2018). Alternatively, the SD sequence on a toxin transcript may bind ‘too perfectly’ to the anti-SD of the 16S rRNA of the 30s ribosomal subunit leading to ribosomal pausing, slower release of the ribosome and thus, a reduced rate of translation (**Figure 7**) (Daou-Chabo et al., 2009; Durand et al., 2012a; Masachis and Darfeuille, 2018).

### Functions of Type I Toxin Antitoxin Systems in Pathogenic Bacteria

Originally discovered in 1983 and characterized as plasmid addiction modules, the increased recognition and characterization of TA systems on the chromosomes of bacteria and archaea has revealed their involvement in several other important processes (Gerdes et al., 1986a; Gerdes et al., 1986b; Thisted et al., 1994; Lee and Lee, 2016; Harms et al., 2018). Currently, bacterial Type I TA systems are hypothesized to function in numerous processes including plasmid maintenance, phage abortive infection, chromosome stabilization, persistence, and virulence (Gerdes, 2016; Kędzierska and Hayes, 2016; Sierra et al., 2019; Peltier et al., 2020). While it is clear that Type I TA systems play an important role in the physiology and virulence of many bacterial species, much is left to learn about these diverse systems (Wang and Wood, 2011; Germain-Amiot et al., 2019; Ma et al., 2019; Song and Wood, 2020a).

#### Plasmid Addiction/Post Segregational Killing

The most well understood role of plasmid encoded Type I TA systems in bacteria is for plasmid addiction. This function ensures that the plasmids on which the TA system is encoded are preserved in a population of actively growing bacteria, through post-segregational killing of any daughter cell lacking the plasmid (Gerdes et al., 1986a; Thisted et al., 1994). Due to the relative stability of the toxin as compared to that of the antitoxin, daughter cells lacking the plasmid on which the TA system is encoded become exposed to the lethal effect of the stable toxin. The role of bacterial TA systems in plasmid addiction was originally characterized with the study of the *E. coli* Hok/Sok Type I TA system, a system encoded on the R1 plasmid (Gerdes et al., 1986b; Gerdes et al., 1986a; Thisted et al., 1994). A similar function was subsequently observed for the Fst/RNAII Type I TA system present on the paDI plasmid of *E. faecalis* (Weaver et al., 1996; Greenfield et al., 2001; Weaver et al., 2009).

#### Persister State Formation

Persister state formation is observed in many bacterial species. In this phenomenon, a heterogeneous population of phenotypically variant bacteria is formed in which metabolism and growth are arrested in a subpopulation, making them resistant to killing by antibiotics (Brauner et al., 2016). This metabolic halt is relieved when antibiotic is removed from the environment, and the persister cells propagate to reestablish the population (Michiels et al., 2016). This bet-hedging strategy may facilitate recurrent and chronic bacterial infections (Fauvart et al., 2011). Known to be controlled by a variety of transcriptional factors and global regulators, several studies now suggest a role for chromosomally encoded TA systems in persister cell formation (Wang and Wood, 2011; Gerdes, 2016; Page and Peti, 2016; Berghoff and Wagner, 2017). For instance, the SOS-dependent TisB/IstR Type I TA system of *Bacillus subtilis* has been shown to play a role in persistence, as induction of TisB led to increased numbers of ciprofloxacin persisters and a deletion of the entire locus abolished the phenotype (Dörr et al., 2010) This involves the SOS-induced cleavage of the IstRI antitoxin upon DNA damage caused by antibiotic, leaving the TisB toxin to damage the *E. coli* membrane, decrease bacterial transcription and translation, and by doing so reduce metabolism and increase antibiotic tolerance (Unoson and Wagner, 2008). Additionally, a recent report demonstrated that overexpression of a chromosomally encoded Hok homologue, HokB in *E. coli* strain BW25113, facilitates persister cell formation through pore induced-ATP leakage and loss of electrochemical potential (Verstraeten et al., 2015; Wilmaerts et al., 2018). Interestingly, for the first time, resuscitation of a Type I TA-induced persister state was demonstrated in a follow up study on HokB, and was shown to occur via two main processes (Wilmaerts et al., 2019). Firstly, the pores induced by HokB dimers are destabilized upon disassembly of the toxin by an DsbC oxidoreductase, and the resulting monomers degraded by the DegQ protease (Wilmaerts et al., 2019). Secondly, the ATP-depleted cells can be replenished through membrane repolarization by Complex I of the Electron Transport Chain (Wilmaerts et al., 2019). Adding to the Type I TA-induced persistence repertoire, the Type I AapAI toxin of *H. pylori* has been shown to transform the bacterium from a spiral to a coccoid *in vitro*, a phenotype which coincides with infections that are resistant to antibiotic-mediated killing. Finally, through an unknown mechanism, the RalR/RalA Type I TA system in *E. coli* was shown to confer protection to Fosfomycin, a broad-spectrum antibiotic (Guo et al., 2014). It is important to note however, that several studies have questioned the direct involvement of TA systems with persister cell formation. According to one hypothesis, the TA-induced persistence phenotype is primarily due to reduced metabolism and not to toxin mediated loss of ATP or ppGpp as some studies suggest (Pontes and Groisman, 2019). These issues, together with other ‘persistent persister misperceptions’ are reviewed in Kim and Wood (2016). Clearly, further studies are required to elucidate the exact molecular mechanisms of persister cell formation and the role that Type I TA systems play in these processes.

#### Chromosome Stabilization

In a similar fashion to plasmid addiction, Type I TA systems have been suggested to play a role in stabilizing portions of the chromosome (Durand et al., 2012b, 1). Several *B. subtilis* Type I TAs are located near prophage regions within the chromosome and are predicted to protect these regions from being deleted (Durand et al., 2012b; Brantl and Müller, 2019). For example, the BsrG/SR4 and YonT/SR6 Type I TA systems in *B. subtilis* are both found on the SPβ prophage element on the chromosome and are suggested to play a role in its stabilization (Reif et al., 2018; Brantl and Müller, 2019). The SPβ prophage element contains genes which may aid *B. subtilis* in survival, including protection from UV light and from the SP10 bacteriophage (Wen and Fozo, 2014). Other *B. subtilis* Type I TA systems whose function might be linked to chromosome stabilization are the TxpA/RatA and BsrH/as-BsrH system. These systems are encoded within *B. subtilis skin* element, a chromosomal region crucial for sporulation (Silvaggi et al., 2005; Wen and Fozo, 2014). Finally, the proximity of Type I TA systems to CRISPR modules (Maikova et al., 2018)and prophage regions (Peltier et al., 2020) in *C. difficile* is suggestive of a role in the chromosome stabilization of the bacterium’s adaptive immunity against invading phages (Soutourina, 2019).

#### Virulence and Defense Against Competitors

Recently, the direct or indirect impact of TA systems, especially Type II TA systems, on virulence of different pathogens has been supported by experimental analyses. The presence of TA systems on virulence-associated plasmids could contribute to virulence by ensuring maintenance of these plasmids, as studies of Type II TA systems have shown (Brendler et al., 2004; Lobato-Márquez et al., 2016b; McVicker and Tang, 2016). Additionally, several chromosomally encoded Type I TA systems have been shown to contribute to bacterial virulence in a variety of ways. The SprA1/SprA1_AS_ and SprA2/SprA2_AS_ Type I TA systems in *S. aureus* encode the toxins, PepA1 and PepA2 respectively, that differ slightly in function (Sayed et al., 2012a; Sayed et al., 2012b; Bronsard et al., 2017; Germain-Amiot et al., 2019). Whereas both toxins can target human polymorphonuclear neutrophils and erythrocytes, PepA1 is ten times more cytotoxic than PepA2 and is suggested to play a role in inter-bacterial competition (Germain-Amiot et al., 2019). Once secreted, both toxins can act together to destroy erythrocytes and competing bacteria. It is hypothesized that destruction of surrounding cells aids in the spread of the *S. aureus* within the host (Germain-Amiot et al., 2019). Another *S. aureus* Type I TA system which likely impacts virulence is the *sprG1*/*sprF1* system (Riffaud et al., 2019, 1). Here, the toxin gene encodes two small toxins of unequal lengths which are secreted extracellularly. Once secreted both toxins can lyse human host erythrocytes, with the longer peptide doing so more efficiently. These toxins are also involved in interbacterial competition where, in this case, the shorter peptide is more effective (Riffaud et al., 2019). Additionally, the ability of a *Salmonella* Typhimurium Type I TA system homologous to the *E. coli hok-sok*, *ldrA-rdl* and *tisB-istR* systems to aid in the intracellular lifestyle of this pathogen, suggests a role in virulence (Lobato-Márquez et al., 2015). Interestingly, deleting the antitoxin of the *E. faecalis* Fst/RNAII Type I TA system, results in hypervirulence in mice and larvae models, increased resistance to oxidative and acid stress, and higher survival rates in macrophages as compared to the wild type strain (Michaux et al., 2014).

#### Defense Against Phages

Type I TA systems have been suggested to defend bacteria from phage attack. This phenotype was first identified in *E. coli* where the Hok/Sok Type I TA system was shown to prevent the T4 phage from spreading throughout the bacteria population through a mechanism termed altruistic killing (Pecota and Wood, 1996). According to this hypothesis, *E. coli* inhibits T4 phage by downregulating the *Sok* antitoxin within a subpopulation of phage infected cells, thus releasing the Hok toxin to induce apoptosis (Pecota and Wood, 1996). Despite such evidence to support the role of TA systems in phage resistance via the killing of infected cells, the lack of direct evident to demonstrate Hok-induced killing somewhat weakens this hypothesis. A recent alternative hypothesis suggests that TA-mediated phage resistance results from a general reduction in metabolism caused by production of the toxin, rather than direct killing of the infected cell by the toxin (Song and Wood, 2018).

### Type I TA Systems in Selected Pathogenic Bacteria

#### Enterococcus faecalis

Aside from *S. aureus*, another pathogen of the ESKAPE group mentioned in this review is *E. faecalis* (Rice, 2008). Though the genus *Enterococcus* has approximately about forty species, the two mostly associated with human disease are *E. faecium* and *E. faecalis* (Van Tyne et al., 2013). Commensals in the gastrointestinal tract, these organisms can cause infection when carried to different sites (Kristich et al., 2014). Additionally, *E. faecalis* is associated with a number of nosocomial infections such as surgical wound infections, sepsis, urinary tract infections and endocarditis (Kau et al., 2005).

Though a number of TA systems have been described for *E. faecalis*, the only Type I TA system described in this pathogen is the *par* locus (Weaver, 2012). Discovered in 1993 on the pAD1 plasmid, *E. faecalis par* locus was the first Type I TA system described in a Gram-positive bacterium and is now a well-known plasmid addiction module (Weaver et al., 1993). The existence of additional copies of the *par* TA system on the *E. faecalis* chromosome, as well as that of *Streptococcus mutans* (reviewed in Weaver et al., 2009; Koyanagi and Lévesque, 2013), *Lactobacillus casei* (Weaver et al., 2009) and*, Lactobacillus rhamnosus* (Weaver et al., 2009; Folli et al., 2017; Maggi et al., 2019) suggests additional functions for this system (Kwong et al., 2010).

The *par* locus consists of two convergently encoded genes; RNAI which encodes for the Fst toxin, and RNAII, which encodes an antisense sRNA antitoxin that represses Fst translation. Both RNAI and RNAII share a common bidirectional terminator (Greenfield et al., 2001). Also, at the 5’ end of each toxin and antitoxin transcript is a direct repeat sequence, DRa and DRb respectively (Greenfield and Weaver, 2000; Greenfield et al., 2000; Greenfield et al., 2001). These direct repeats and the common bidirectional terminator sequence constitute the base pairing interaction regions between the antitoxin and the toxin transcript. The Fst toxin is the founding member of a large superfamily of toxins. A Fst-like toxin is typically a 33 amino acid protein, consisting of a transmembrane domain and a charged C-terminal tail, both of which are essential for toxin activity. Fst-like toxins cause nucleoid condensation within the pathogen, leading to septal displacement and disproportional division of DNA into daughter cells (Maggi et al., 2019). Göbl et al., 2010 also suggested that the lethal activity of the Fst toxin is mediated by insertion of the alpha helical hydrophobic domain into the bacterial membrane, an activity which affects membrane permeability.

#### Streptococcus mutans

*S. mutans* is a Gram-positive facultative anaerobe that, together with other streptococcal species, is associated with the human oral cavity, pharynx and intestine (Loesche, 1986). *S. mutans* is associated with dental caries in humans as well as infections of the muscle, skin, cardiovascular and nervous systems (Forssten et al., 2010).

The *S. mutans* chromosomally encoded Fst-like toxin, Fst-Sm, together with its associated cis-encoded sRNA antitoxin called *srSm* constitute the first reported functional Type I TA system in the genus *Streptococcus* (Koyanagi and Lévesque, 2013). A homologue of *E. faecalis* Fst toxin, Fst-Sm was an unannotated open reading frame found within IGR176 intergenic region of the *S. mutans* UA159 reference strain (Fozo et al., 2010). Convergently encoded at the 3’ end of *fst-Sm* is the antitoxin sRNA, *srSm*, which is suggested to block Fst-Sm expression by binding to a tandem repeat close to the translation initiation region of the toxin transcript. Fst-Sm is reported to encode for a small hydrophobic peptide, the overexpression of which results in growth inhibition of *S. mutans*. Counterintuitively, the ectopic expression of the entire Fst-Sm/srSm Type I TA locus reduced, rather than increased, the number of cell-wall tolerant persisters. Further experimental validation is required to fully understand the role that the Fts-Sm/srSm Type I TA system plays in the virulence and physiology of *S. mutans*.

#### Bacillus spp.

*Bacillus* species are Gram-positive bacilli which, much like *Clostridia*, are known for their spore forming ability that enables them to survive harsh environmental conditions. Composed of approximately 200 members, *Bacillus* is a large genus consisting of both pathogenic and non-pathogenic species. *B anthracis* and *B. cereus* are associated with anthrax in livestock and food poisoning in humans, respectively. Of the more than a dozen Type I TA systems identified in *B. subtilis*, the TxpA/RatA (Silvaggi et al., 2005; Durand et al., 2012a), BsrG/SR4 (Jahn et al., 2012), bsrE/SR5 (Müller et al., 2016) and yonT/yoyJ/SR6 (Reif et al., 2018) systems are the most extensively characterized to date.

Through microarray work that sought to identify novel untranslated RNAs within the *B. subtilis* genome in 2005, the first Type I TA system in *Bacillus*, *txpA/*RatA was identified, and shown to be located on chromosomal *skin* element (Silvaggi et al., 2005). This consists of the 59 amino acid long toxin and a convergently encoded antitoxin, RatA. Unlike BsrG/SR4, binding of the RatA sRNA antitoxin to the TxpA toxin transcript does not induce translation inhibition (Silvaggi et al., 2005). Instead, the RatA sRNA antitoxin interacts with the toxin’s mRNA at the 3’ end via an extensive amount of nucleic acid complementarity forming an RNA duplex which is cleavable by RNAse III. Similar to BsrG/SR4, deletion of *ratA* antitoxin caused lysis of *B. subtilis* on agar plates, but only after 5 days of incubation (Silvaggi et al., 2005). Aside from RatA repression, another possible mechanism by which *B. subtilis* is protected from the toxin gene is through an unusually strong (11-12) base pairing between the TxpA SD sequence and the anti-SD 3’ end of 16S rRNA (Daou-Chabo et al., 2009; Durand et al., 2012a). This near perfect interaction could potentially lead to ribosomal pause and slow translation as reviewed above (Daou-Chabo et al., 2009; Durand et al., 2012a). TxpA/RatA, is distinct from BsrG/SR4 in that RNAse III is indispensable in TxpA repression unlike in BsrG, as *rnc* mutants (lacking RNAse III) showed lysis of *B. subtilis*, even in the presence of the RatA (Silvaggi et al., 2005; Durand et al., 2012a; Jahn et al., 2012; Brantl and Müller, 2019). Since its characterization, no work has been done on elucidating the exact mechanism of TxpA activity, though with its predicted N-terminal transmembrane domain, it is likely a membrane targeting toxin like many Toxins of Type I TA systems (Silvaggi et al., 2005; Fozo et al., 2008b)

The *bsrG* **/** *sr4* loci encodes the first *B. subtillis* temperature dependent Type I TA system described (Jahn et al., 2012; Jahn et al., 2015). This locus consists of *bsrG*, a gene encoding a small hydrophobic peptide, and *sr4*, a gene encoding an sRNA antitoxin that functions to repress production of the BsrG toxin. Having a predicted transmembrane domain and charged C-terminus, BsrG was shown to localize to the *B. subtilis* membrane (Jahn et al., 2015). However, unlike many Type I TA toxins, BsrG does not perforate the cell membrane, but rather disturbs envelope biosynthesis which leads to cell membrane invagination, dislocation of the cell wall synthesis machinery, and ultimately cell lysis (Jahn et al., 2015). Cognate toxin repression by SR4 antitoxin is mediated by an extensive amount of nucleic acid complementarity to the toxin transcript. The BsrG/SR4 system is unique in that toxin repression is dependent on both translation inhibition and degradation of the toxin transcript. Upon SR4 binding to the 3’ end of the *bsrG* transcript, structural changes occur in the area around the SD that inhibit translation. Additionally, the mRNA-sRNA duplex attracts an RNase for cleavage. In the steady state, antitoxin abundance is higher than that of the toxin due to the antitoxin promoter having 6-10-fold higher strength. At 48°C, the half-life of the *bsrG* mRNA decreases, resulting in significantly lower levels of the toxin at that temperature (Jahn et al., 2012).

The *txpA/*RatA Type I TA system, located on *B. subtilis* chromosomal *skin* element, was the first Type I TA system described in this species (Silvaggi et al., 2005; Durand et al., 2012a.) The RatA sRNA antitoxin interacts with the 3’ end of the toxin encoding *txpA* transcript via an extensive amount of nucleic acid complementarity, forming an RNA duplex which is cleavable by RNAse III.

A multi-stress responsive module BsrE/SR5 is another *B. subtilis* Type I TA and is located on the P6 prophage on the *B. subtilis* chromosome (Irnov et al., 2010; Durand et al., 2012b; Müller et al., 2016). One major characteristic of this module is the susceptibility of the toxin and antitoxin transcripts to RNAse degradation by a variety of ribonucleases, in response to different kinds of stress. Under O_2_, acidic and iron stress, the antitoxin SR5 is degraded by endoribonuclease J1. On the other hand, J1 degradation of *bsrE* transcript occurs in the presence of temperature shock and alkaline stress. Both transcripts however are rapidly degraded by endoribonuclease Y under ethanol stress. Whereas the antitoxin promoter strength is 10-fold more than that of the toxin, the toxin transcript is also about 10-fold more stable than its cognate antitoxin. The antitoxin transcript is cleaved by the exonuclease PnP prior to binding to the target toxin transcript.

#### Staphylococcus aureus

*S. aureus* colonizes the skin and mucous membranes of approximately a third of the world’s population (Williams, 1963; Otto, 2010). Though it exists on human skins and nares as normal flora, *S. aureus* can cause a variety of infections upon entry into the bloodstream or deeper tissues of the body. *S. aureus* is the etiologic agent of several human diseases ranging from relatively mild skin and soft tissue infections, to life threatening infections such as necrotizing fasciitis, empyema, meningitis, and sepsis (Kobayashi et al., 2015). *S. aureus* is armed with several virulence factors that aid in survival within their human host (Otto, 2010), including five Type I and three Type III TA systems (Schuster and Bertram, 2016a; Harms et al., 2018; Habib et al., 2020).A homologue of *E. faecalis* Fst/RNAII, SprA1-SprA1_AS_ was the first Type I TA system discovered in *S. aureus*. This TA system is encoded within the SaPIn3 pathogenicity island of the Newman strain (Pichon and Felden, 2005; Sayed et al., 2012a; Schuster and Bertram, 2016b; Bronsard et al., 2017; Habib et al., 2020). Subsequent analyses revealed a homologous locus termed SprA2/SprA2_A_s (Germain-Amiot et al., 2019). This second system is located within the same *S. aureus* pathogenicity island, and shares 75% sequence similarity with the previously characterized SprA1/SprA1_AS_ TA system (Germain-Amiot et al., 2019). As with Type I TA systems in other Gram-positive bacteria, genes encoding the toxin and antitoxin components of the SprA/SprA_AS_ system are encoded antisense to each other and overlap at their 3’ ends, leading to perfect complementarity between these portions of the toxin mRNA and the antitoxin sRNA (Sayed et al., 2012a; Germain-Amiot et al., 2019). Experimental characterization of the SprA2/SprA2_AS_ system revealed however, that the functional interaction between the antitoxin sRNA and the toxin mRNA occurs via the 5’ end of each molecule. This interaction abolishes toxin translation by blocking the ribosomal binding site and impeding ribosome loading (Habib et al., 2020).

Despite the high level of similarity, cross-regulation experiments demonstrated that between the *sprA1*/SprA1_AS_ and *sprA2*/SprA2_AS_ systems, antitoxin regulation is specific, each antitoxin only regulating production of its cognate toxin (Germain-Amiot et al., 2019).

The *sprA1*/SprA1_AS_ and *sprA2*/SprA2_AS_ system both encode for short toxic peptides, PepA1 and PepA2 (Sayed et al., 2012b; Germain-Amiot et al., 2019). While both PepA1 and PepA2 have been shown to be cytotoxic against human neutrophils, (Sayed et al., 2012a; Sayed et al., 2012b; Germain-Amiot et al., 2019), functional differences between these proteins have been identified. Firstly, unlike PepA2, PepA1 has been shown to play a role in interbacterial competition. Also, whereas osmotic shock and stringent conditions trigger the production of PepA2, PepA1 production is stimulated by acidic and oxidative stress. Such findings suggest differential production and different roles of these two Type I TA systems in the physiology and virulence of *S. aureus* (Germain-Amiot et al., 2019).

Another *S. aureus* Type I TA system, *sprG1/SprF1*, was discovered on the ΦSa3 PI mobile genetic element (Riffaud et al., 2019). This Type I TA locus is a homologue of the TxpA-RatA system in *B. subtilis* (Fozo et al., 2010) An additional 3 copies of this system, designated *sprG2*-4/SprF2-SprF4, were later identified on the *S. aureus* core genome (Riffaud et al., 2019). The toxin gene *sprG1* encodes two peptides of unequal lengths utilizing different translational start sites on the transcript (Pinel-Marie et al., 2014; Wen and Fozo, 2014; Schuster and Bertram, 2016b). In addition to being self-lethal to *S. aureus*, these pore forming toxins have been shown to be capable of lysing both human red blood cells and competing bacterial species (Pinel-Marie et al., 2014). *sprG1* shares complementarity at its 3’ end with SprF1, a relatively unstable sRNA antitoxin whose constitutive expression represses the production of the SprG1 toxin. Toxins from the *sprG2*-SprF2, *sprG3*-SprF3, *sprG4*-*SprF4* TA systems also mediate bacteriostatic action to different extents in *S. aureus*, with a significant level of cross talk amongst antitoxin-mediated regulation (Riffaud et al., 2019; Riffaud et al., 2020).

#### Helicobacter pylori

*H. pylori* is a Gram-negative spiral bacterium that colonizes fifty percent of the human population. *H. pylori* is the first direct link between bacteria and cancer (gastric cancer), as well as the major cause of other gastric diseases such as peptic ulcer and gastritis (Kusters et al., 2006). With the site of infection being the human stomach, *H. pylori* possesses diverse molecular mechanisms that aid in survival within that environment (Wroblewski et al., 2010).

*H. pylori* carries six chromosomally encoded TA systems, each encoding a small toxin designated AapA_1-6_ and a cis encoded antisense sRNA, designated IsoA_1-6_. These systems were identified by the analysis of transcriptomic data, and were found to be expressed at high levels during the exponential phase of growth (Sharma et al., 2010). Of these TA systems, two (AapA1/IsoA1 and AapA3/IsoA3) were predicted to be members of the Type I TA family due to the arrangement of the genes within the loci, and the fact that production of the toxin is inhibited by the cognate antisense RNA (Sharma et al., 2010; Arnion et al., 2017). Further studies in *H. pylori* confirmed that the *aapA1/isoA1* locus encodes for a typical Type I TA system, with the translation of the AapA1 peptide leading to reduced growth and lysis of the bacterium (Arnion et al., 2017). Interestingly, the full length AapA1 mRNA is translationally inactive upon transcription, as the SD is sequestered by a long-distance interaction (LDI) between the 5’ and 3’ ends of the transcript (Arnion et al., 2017). This structure gives the full-length transcript increased stability, not only preventing translation but also preventing interaction with the antitoxin. Processing of the *aapA* transcript at the 3’ end, induces structural alterations within the 5’ UTR that result in exposure of the SD sequence and translation of the peptide. This 3’ processing also allows for the IsaA1 antitoxin to interact with the *aapA1* transcript through two kissing loop interactions, forming an RNA duplex that is subsequently cleavable by RNAse III (Arnion et al., 2017).

In addition to the LDI mediated prevention of toxin production, subsequent analyses of the *H. pylori* AapA3/IsoA3 TA system demonstrated that premature toxin translation is prevented by the formation of transiently stable stem loop structures within the 5’ UTR and the toxin open reading frame that is capable of sequestering the SD sequence within the growing transcript (Masachis et al., 2019). Such a mechanism prevents ribosomal loading and toxin translation before the 3’ end of the transcript is generated by progression of the RNA polymerase.

#### Clostridium difficile

*C. difficile* is the major cause of antibiotic resistant, hospital acquired diarrhea (Heinlen and Ballard, 2010). Resistance to stressful conditions, and the ability to reestablish infection within treated hosts is due largely to spore formation by this pathogen. Additionally, TA system may contribute to the pathogen’s survival under adverse conditions (Vedantam et al., 2012).

Compared to those of Type II TA systems, Type I TA systems are relatively difficult to identify (Lobato-Márquez et al., 2015; Coray et al., 2017). Homology searches using previously characterized Type I TA systems from other bacteria did not identify many candidate Type I TAs in *C. difficile* (Soutourina, 2019). However, RNAseq-based promoter mapping and subsequent *in silico* analyses identified 251 putative sRNAs in *C. difficile* (Soutourina et al., 2013). This finding facilitated further data mining in the search for transcripts antisense to, or overlapping with genes that encode for small proteins of unknown function (Maikova et al., 2018). From this, six TA candidates were identified and three of them, CD2517.1-RCd8, CD2907.1-RCd9, and CD0956.2-RCd10, were studied further (Maikova et al., 2018). The identified putative toxins are predicted to be 50 amino acid long transmembrane proteins with conserved charged amino acids at the C-terminus. Production of these toxin proteins arrest *C. difficile* growth, a phenotype which is inhibited by cognate sRNA antitoxins with high specificity (Maikova et al., 2018). Interestingly, a close association of these TA systems to CRSPR sequences is observed, suggesting a possible role in maintenance of these important regions of the bacterial chromosomal (Maikova et al., 2018).

#### Escherichia spp.

*E coli* is a Gram-negative rod-shaped bacterium. Though a major constituent of normal intestinal flora, more than six pathotypes have been noted to cause gastrointestinal and urinary tract infections. *E. coli* is one of the most extensively studied bacteria for Type I TA systems. In total, approximately 26 Type 1 TA systems have been identified in both pathogenic and non-pathogenic strains of *E. coli* (Fozo et al., 2010). Selected systems will be highlighted here.

Apart from the *E. faecalis* Fst/RNAII system, the early days of bacteria TA research saw the discovery of the plasmid encoded Hok/Sok Type I TA system within *E. coli*, now one of the most studied Type I TA system in this species (Gerdes and Wagner, 2007; Yang and Walsh, 2017). A plasmid addiction module, Hok/Sok is involved in maintaining the R1 plasmid through post segregational killing, a phenomenon largely adapted to explain the roles of plasmid encoded TA systems within bacteria (Gerdes et al., 1986b; Gerdes et al., 1986a). The Hok protein is a transmembrane protein that has been shown to target the bacterial membrane, destroying its integrity through the formation of tiny anion selective pores that result in the depletion of ATP and the formation of so called ‘ghost cells’ (Gerdes et al., 1986b; Peng et al., 2019). The Sok antitoxin is an sRNA that is involved in the indirect inhibition of Hok translation, by blocking an open reading frame, *mok*, that overlaps with the *hok* sequence, whose translation is coupled to *hok* translation. Importantly, for either *mok* translation or Sok inhibitory binding to occur, the full length *hok* mRNA must first undergo 3’ end processing to release the transcript from a fold back inhibition mediated by binding between the 5’ and 3’ ends of the transcript (Thisted et al., 1994).

Like the Hok/Sok system is the Ldr/Rdl putative Type I TA system (Kawano, 2012). This system is composed of a short LdrD toxin whose production in *E. coli* is lethal to the organism and whose production is regulated by the sRNA antitoxin, RdlD. Much like the Hok/Sok/Mok arrangement, the *ldrD* toxin transcript has a small open reading frame, *ldrX* within its long 5’ UTR which overlaps with the translation initiation region of the toxin gene. Hence translation of the toxin gene is coupled to that of the small open reading frame *ldrX*. RdlD inhibits the production of LdrD by blocking the translation of *ldrX* (Kawano et al., 2002; Kawano, 2012).

Another *E. coli* Type I TA system is the SymE/SymR system (Kawano et al., 2007). This system is composed of an endonuclease toxin, SymE, and a sRNA antitoxin, SymR. Unlike most Type I antitoxins, SymR is unique in having a higher stability than its toxin counterpart. At steady state, *symE* expression is tightly repressed by three different processes. Firstly, the *symE* gene is transcriptionally regulated by the LexA repressor. Second, following transcription, the SymR antitoxin prevents translation from the *symE* transcript through complementary base pairing in the 5’ UTR of the toxin transcript. Finally, Lon protease acts post-translationally to degrade the SymE toxin (Kawano, 2012). This multilayer repression of the toxin likely protects the bacteria from toxin production until it is needed, such as when the bacterium experiences DNA damage. Under such circumstances, production of the SymR antitoxin is repressed by the SOS response, allowing toxin production to proceed. Once produced, SymE inhibits growth by degrading DNA and decreasing protein synthesis (Kawano et al., 2007, Kawano, 2012).

Another well studied Type I TA system in *E. coli* is TisB/IstR system. This is another SOS-induced Type I TA toxin like SymE/SymR (Kawano et al., 2007). As an SOS-induced toxin gene, *tisB* is repressed in the absence of stress by the master regulator, LexA. In addition, TisB production is repressed by a trans encoded sRNA antitoxin, IstR. Unique among Type I TA systems, *tisB* expression is inhibited by a structure in its 5’ UTR that occludes a ribosome standby site (RSS) on the transcript. Like most Type I TA toxin transcripts, a processing event is required to render *tisB* translatable. This processed transcript, however, can also be bound by the antitoxin IstR-1, to generate a RNA duplex that is cleaved by RNAse III (Vogel et al., 2004). In a IstR-1 deleted strain, TisB is translated and causes growth inhibition due to membrane depolarization.

Present on the chromosome of *E. coli* O157:H7 are two nearly identical Type I TA systems designated *zorO/orzO* and *zorP/orzP* (Wen and Fozo, 2013). Of these two systems, *zorO/orzo* has been studied in more detail (Wen and Fozo, 2013). Increased production of ZorO is toxic to *E. coli*, a phenotype that can be reversed by the coproduction of the OrzO antitoxin in a manner dependent upon RNase III. In addition to repression by ZorO, the full-length ZorO 5’UTR is sufficient to inhibit its own translation by forming an inhibitory structure that sequesters the SD (Wen et al., 2017). OrzP-dependent repression of *zorO* is mediated by nucleic acid complementarity between the sRNA antitoxin and the target toxin transcript. Interestingly, despite nucleic acid sequence similarity between the two antitoxins, repression of *zorO* is achieved only by the OrzO antitoxin and not by neighboring OrzP molecule.

#### Salmonella

*S. enterica* serovar Typhimurium is a member of the Enterobacteriaceae family and has been implicated in a variety of food-borne illnesses. Three *E. coli* Type I TA homologues found in *Salmonella enterica* serovar Typhimurium, *hok-sok*_ST_, *ldrA-rdlA*_ST_, *tisB-istR*_ST_ (described above) have been shown to play a role in the intracellular lifestyle of the pathogen (Lobato-Márquez et al., 2015). As functional Type I TA toxins, overproduction of Hok_ST_, LdrA_ST_ or TisB_ST_ inhibits *S*. Typhimurium growth (Lobato-Márquez et al., 2015). *S*. Typhimurium is known to inhibit its proliferation to survive within fibroblasts (Cano et al., 2001). Consistent with their growth inhibition phenotype, genes encoding these three toxins were shown to be upregulated, in bacteria isolated from fibroblasts. More importantly, deletion of the toxin genes resulted in limited intracellular survival within fibroblasts, based on survival assays, indicating the significance of the toxin genes in survival within such intracellular environments (Lobato-Márquez et al., 2015). Relevance of a TA system in survival of *S.* Typhimurium is not new, as Type II TA modules have previously been suggested in *S.* Typhimurium persistence within macrophages (Stårsta et al., 2020). However, this study highlights for the first time that Type I TAs are equally important in facilitating adaptation of *S*. Typhimurium to the intracellular environment.

Recently, a new Type I TA system has been proposed in *Salmonella*, termed TimR/TimP (Andresen et al., 2020). TimP transcript was originally identified in *E. coli*, through a transcriptomic study as a RNA of unknown function, RyfA, and no conserved open reading frame (ORF) was identified for it in that study (Wassarman et al., 2001; Andresen et al., 2020). Subsequently, conservation within different genomic contexts, and expression of RyfA were observed in other members of the *Enterobacteriaceae* family (Hershberg et al., 2003; Andresen et al., 2020), and relevance in virulence and biofilm formation observed for RyfA expression in *Shigella* (Fris et al., 2017) and ocular pathogenic *E. coli* respectively (Ranjith et al., 2019). However, the work done in *S. enterica* serovar Typhimurium showed the production of small toxic membrane peptide, TimP from previously termed *ryfA* sRNA. Translation of this toxin was shown to be repressed by a divergently encoded sRNA, TimR. When overexpressed, TimP damages the membrane, leading to growth inhibition of *Salmonella* (Andresen et al., 2020).

## Section 2: Type III Toxin Antitoxin Systems

### Functions of Type III Toxin Antitoxin Systems in Pathogenic Bacteria

Like Type I TA systems, Type III TA systems have a protein toxin and an sRNA antitoxin. Unlike Type I TA systems, however, the Type III sRNA antitoxin functions to protect against the lethal activity of the toxin by directly binding the toxin itself. The locus encoding each Type III TA system has a single promoter which constitutively drives the transcription of a polycistronic transcript encoding for both the toxin protein and antitoxin sRNA. The antitoxin gene is encoded upstream that of the toxin, with a Rho-independent transcriptional terminator separating them. This Rho-independent terminator is suggested to control the sRNA to toxin mRNA stoichiometry, allowing excess of antitoxins via infrequent read-throughs. The antitoxin transcript is 200-nucleotide long when initially transcribed, and it is characterized by the presence of nearly identical tandemly arranged repeat sequences. The number of repeat sequences is specific for each Type III TA system described. Each repetitive sequence is 36 nucleotides long and can be separated by cleavage by the toxin which is an endonuclease. Following cleavage, the antitoxin fragments form a complex with the endonuclease toxin that functionally, inactivates the toxin. Such inactivation prevents the toxin from cleaving other essential cellular targets (Blower et al., 2012; Brantl and Jahn, 2015).

### Type III TA Systems in Pathogenic Bacteria

#### Pectobacterium atroseptica

Research on Type III TA systems is limited, with just one such system identified in a pathogenic bacterium to date; the ToxI/ToxN system in the plant pathogen, *Pectobacterium atroseptica.* (Short et al., 2018). Here, the plasmid encoded Type III TA locus consists of the ToxN toxin and the ToxI antitoxin.

While the best studied Type III TA system is the ToxI/ToxN system in the plant pathogen, *Pectobacterium atroseptica* (Short et al., 2018), other type III TA systems have been studied based on similarity to this system. In Lactobacillus lactis, the plasmid encoded AbiQ/AntiQ TA system is similar in genetic context; having a long antitoxin transcript at the 5’ end of a polycistronic transcript separated from the gene encoding the endonuclease toxin by a weak terminator (Samson et al., 2013). Two other Type III systems, TenpN/TenpI and CptN/CptI, have been predicted based on homology to the amino acid sequence of ToxN. Together with the well characterized ToxI/ToxN module, these make up three families of Type III TA systems (Blower et al., 2012).

## Closing Remarks

Despite the recent discoveries of new Type I TA systems in bacteria, the number of RNA-regulated TA systems identified and verified to date still trails behind that of the Type II TA system. This could be due in part to the relatively small size of Type I toxin proteins, and to the fact that regulatory sRNAs can be difficult to identify by sequence analyses. Additionally, the toxins produced by Type I TA systems do not share extensive conservation with each other but rather only share the characteristic transmembrane domain and a C-terminus made of aromatic amino acids. This results in many Type I TA systems having sequence divergence across different bacteria. In fact, Fozo et al. (2010) observed the difficulty in predicting Type I TA systems using the default parameters of the NCBI homology search platforms alone. However, by employing a computational approach involving an exhaustive TBLASTN and PSI-BLAST across approximately 800 sequenced genomes, the group demonstrated that, contrary to what was previously reported, Type I TA systems are not narrowly distributed within bacteria by horizontal transfer, but rather are widely distributed across different bacteria (Fozo et al., 2010). More recently this approach was used to identify 5 Type I TA homologs in *Salmonella* Typhimurium strain SL1344 (Lobato-Márquez et al., 2015). Additionally, the study increased the known number of Type I TA systems by discovering new loci using search parameters based on other known Type I TAs, including location of the short open reading frames in the intergenic region, the presence of a transmembrane domain and a bulky C-terminus residue, and the proximity of the putative antitoxin sRNA (Fozo et al., 2010).

The identification of new Type III TA systems, on the other hand, has highly relied on homology searches of structural motifs across different bacteria, leading to the discovery of new putative Type III TA homologues though many lack experimental validation to date. Future investigations will no doubt, provide additional information about the distribution and function of Type III TA systems in pathogenic bacteria (Blower et al., 2012).

A recent finding suggests the existence of a new class of TA systems, one in which both the toxin and antitoxin are sRNA molecules. The inaugural member of this new class of TA systems is the SdsR/RyeA in *E. coli* (Choi et al., 2018). Here, the RyeA toxin targets and represses expression of the *tolC* and *mutS* genes in an Hfq-dependent manner, leading to cell lysis of the bacterium. Interestingly, a previously characterized *E. coli* sRNA has recently been shown to encode for a small peptide in *Salmonella* and is now suggested to be a part of a Type I TA pair (Andresen et al., 2020). Together, these findings therefore present the interesting possibility of a Type I TA toxin having regulatory function as both an sRNA and a protein. Similarly, an interesting question was raised in a recent review about the possibility of Type I and III sRNA antitoxins having additional regulatory activity within bacteria, aside from toxin repression. Dual functionality of components of bacterial TA systems is clearly worthy of further investigating (Riffaud et al., 2020).

Different studies assign different functions to various Type I TA systems. Deletion experiments, overexpression experiments and identifying the conditions in which the toxins are expressed provide clues about the putative role(s) of a TA system. However, a number of these findings are lacking in their universality and are not fully confirmed. A possible explanation for the difficulty in functional characterization of the sRNA-regulated TA systems is the reliance on overexpression experiments, which might not represent the physiological levels of the molecules in the bacterium (Masachis et al., 2019).

In conclusion, the RNA regulated TA systems provide additional evidence to the importance of regulatory RNAs in controlling bacteria gene expression. As with all sRNA-mediated regulation, the involvement of a sRNA antitoxin in a TA system could provide an advantage over a protein counterpart. The extra energy investment required for the production of a protein antitoxin is relieved if that antitoxin is instead, an sRNA molecule (Fozo et al., 2008b). There is no doubt that with further investigation the recognized significance of the role that RNA regulated TA systems play in the physiology and virulence of pathogenic bacteria will continue to grow as will the knowledge of the molecular mechanism underlying their activity.

## Author Contributions

DS wrote the first draft which was contributed to, and edited, by EM. All authors contributed to the article and approved the submitted version.

## Funding

NIH (R15AI103887-01A1) - supported studies related to this paper. Ohio University Heritage College of Osteopathic Medicine: Will help cover the publication costs. Infectious and Tropical Disease Institute, Ohio University: Will help cover the publication costs. Molecular and Cellular Biology Program, Ohio University: Will help cover the publication costs.

## Conflict of Interest

The authors declare that the research was conducted in the absence of any commercial or financial relationships that could be construed as a potential conflict of interest.
